# Risk Factors and Prognosis of Polymyxin- and Carbapenem-Resistant Enterobacteriaceae Infections: A Propensity-Matched Real-World Study

**DOI:** 10.3390/microorganisms13061256

**Published:** 2025-05-29

**Authors:** Jian Xu, Mei Liang, Yanan Luo, Junyan Qu

**Affiliations:** 1 Center of Infectious Disease, West China Hospital, Sichuan University, 37 Guoxue Lane, Chengdu 610041, China; xujian19880926@gmail.com (J.X.); liangmei1216@163.com (M.L.); lyn08140911@163.com (Y.L.); 2 Department of Infectious Diseases, Santai People’s Hospital, 139 Jiefang Xia Street, Mianyang 621000, China

**Keywords:** polymyxin, carbapenem-resistant Enterobacteriaceae, risk factors, prognosis

## Abstract

The risk factors and prognosis of polymyxin- and carbapenem-resistant Enterobacteriaceae (PR-CRE) infections were analyzed to reduce their incidence and concurrently improve patient prognosis. This retrospective study analyzed patients with CRE infections admitted to West China Hospital of Sichuan University between 1 September 2019 and 30 September 2023. Based on polymyxin susceptibility, the cases were categorized into PR-CRE and PS-CRE (polymyxin-susceptible CRE) groups, with 1:1 propensity score matching performed between the two cohorts. Comprehensive data, including demographic characteristics, laboratory findings, antibiotic regimens, and clinical outcomes, were collected and analyzed to identify risk factors for PR-CRE infections and evaluate treatment efficacy. This study aims to provide evidence-based references for infection control strategies and antimicrobial stewardship in managing PR-CRE infections. A total of 254 patients were included in this study, with 127 patients in the PR-CRE group. The sensitivity rates of isolates in the PR-CRE group to tigecycline and ceftazidime–avibactam were 94.4% and 88.9%, respectively. Multivariate analysis identified chronic organic disease (OR 2.747, 95% CI 1.303–5.789; *p* = 0.008) and the use of polymyxin ≥ 3 days (OR 19.203, 95% CI 7.126–51.752; *p* < 0.001) as independent risk factors for PR-CRE infection. Moreover, ceftazidime–avibactam-based regimens were superior to tigecycline-based regimens for the treatment of PR-CRE infections (71.43% vs. 58.46%), especially in critically ill patients (33.33% vs. 58.82%). Finally, a SOFA score ≥ 5.5 (HR 6.718, 95% CI 2.526–17.866; *p* < 0.001) was identified as an independent risk factor for 28-day mortality in patients with PR-CRE infection. The presence of chronic organic diseases and the use of polymyxin for ≥3 days were identified as independent risk factors associated with PR-CRE infections in hospitalized patients, highlighting the need to optimize polymyxin use. Furthermore, the efficacy of ceftazidime–avibactam-based regimens may be superior to tigecycline-based regimens for the treatment of PR-CRE infections.

## 1. Introduction

Polymyxins, including polymyxin B and colistin (also referred to as polymyxin E), were first discovered in the late 1940s and were subsequently used for the treatment of Gram-negative bacterial infections in the 1950s [1,2]. Owing to their nephrotoxicity and the ongoing development of novel antimicrobial agents, the clinical use of polymyxins has progressively declined. However, they have been extensively applied as growth promoters and for the prevention and treatment of infections in animal husbandry [3,4]. Since the 20th century, the irrational use of antimicrobial agents has driven bacterial resistance, particularly in the case of carbapenem-resistant Gram-negative bacteria, such as carbapenem-resistant *Enterobacteriaceae* (CRE), carbapenem-resistant *Pseudomonas aeruginosa* (CRPA), and carbapenem-resistant *Acinetobacter baumannii* (CRAB). These bacteria are characterized by their high resistance, limited treatment options, and poor treatment outcomes, posing a serious threat to human health [5]. Consequently, polymyxin has re-emerged as a last-line treatment for severe infections caused by multidrug-resistant (MDR) Gram-negative bacteria in the clinical setting. Nonetheless, there are a growing number of reports on polymyxin resistance, with resistance rates steadily increasing. As a result, treatment options against MDR Gram-negative bacteria are becoming scarce [6,7,8,9,10]. In particular, the emergence of *Enterobacteriaceae* strains resistant to both polymyxins and carbapenems poses significant challenges for clinical treatment. Indeed, polymyxin- and carbapenem-resistant Enterobacteriaceae (PR-CRE) infections might further increase patient mortality [11,12,13]. However, research on the risk factors for PR-CRE infections remains limited, and there are currently no universally accepted international guidelines for the treatment of PR-CRE. Therefore, there is an urgent need to investigate risk factors and prognosis associated with PR-CRE infections to minimize their occurrence, explore novel treatment options, and improve patient prognosis.

## 2. Materials and Methods

### 2.1. Patients and Definitions

This retrospective observational cohort study was conducted in West China Hospital, Sichuan University. Data were retrospectively collected from electronic medical records and analyzed anonymously. The study included patients with CRE infection who were admitted between 1 September 2019 and 30 September 2023. CRE was defined as Enterobacteriaceae that exhibited resistance to carbapenem antibiotics or generated carbapenemase enzymes. PR-CRE was defined as CRE resistant to polymyxins, whereas polymyxin-sensitive CRE (PS-CRE) referred to CRE that remained sensitive to polymyxins.

The inclusion criteria were as follows: (1) patients aged ≥ 18 years; (2) microbiologically confirmed carbapenem-resistant Enterobacteriaceae infection; and (3) presence of clinical manifestations of infection (e.g., fever, sputum production, or urinary tract irritation). In addition, imaging evidence was necessary for the diagnosis of pulmonary infections. Exclusion criteria included (1) pregnancy; (2) cases clinically identified as contamination, colonization, or mixed infections; and (3) insufficient or unavailable data. Cases that met the inclusion criteria were categorized into either the PR-CRE or PS-CRE group based on their polymyxin susceptibility profiles. A total of 127 cases with PR-CRE infections met the inclusion criteria. To minimize potential confounding and ensure group comparability, propensity score matching was performed in a 1:1 ratio, balancing age, sex, and bacterial species between the PR-CRE and PS-CRE groups. As a result, 254 patients were ultimately included in the final analysis. For all enrolled patients, data were collected on age, gender, hospital admission and discharge details, sampling time, comorbidities, chronic organ disease status at sampling sites, history of invasive procedures, and antimicrobial exposure before sampling. In addition, for patients in the PR-CRE group, further data were obtained on post-infection treatment regimens, therapeutic responses, and 28-day survival outcomes.

### 2.2. Efficacy and Prognosis Analysis in Patients with PR-CRE Infection

Patients were divided into three groups: the tigecycline treatment group (including patients treated with tigecycline monotherapy and those treated with tigecycline in combination with other active antibiotics (OAAs) other than ceftazidime–avibactam (CAZ-AVI)); the ceftazidime–avibactam treatment group (including patients treated with ceftazidime–avibactam monotherapy and those treated with ceftazidime–avibactam in combination with OAAs other than tigecycline); and the combination treatment group (including patients treated with both tigecycline and ceftazidime–avibactam).

### 2.3. Laboratory Studies

The laboratory parameters collected included white blood cell (WBC) count, neutrophil count, absolute lymphocyte count, procalcitonin (PCT) level, interleukin-6 (IL-6) level, C-reactive protein (CRP) level, CD3+ T lymphocyte count, CD4+ T lymphocyte count, CD8+ T lymphocyte count, and antimicrobial susceptibility testing (AST) results. Bacterial identification and antimicrobial susceptibility testing (AST) were conducted using the MicroScan WalkAway96 system (Siemens, USA). The minimum inhibitory concentration (MIC) of CAZ-AVI was determined using the Kirby–Bauer method (Zone Diameter ≤ 20 mm for resistance and ≥21 mm for susceptible), and the MIC of polymyxin B was assessed via broth microdilution, according to Clinical and Laboratory Standards Institute (CLSI) guidelines. Polymyxin B breakpoints followed the criteria established by CLSI: ≤2 mg/L for intermediate and ≥4 mg/L for resistance [14]. The MIC reference ranges for the remaining antimicrobial agents are provided in Supplementary Table S1.

### 2.4. Antibiotic Strategies and Clinical Outcomes

Patients with PR-CRE infection received treatment with tigecycline, CAZ-AVI, or combinations of these agents and other susceptible drugs. In addition, age-adjusted Charlson Comorbidity Index (aCCI) and Sequential Organ Failure Assessment (SOFA) scores were calculated, and data on post-infection treatment regimens, treatment effectiveness, and 28-day survival outcomes were collected. Effective treatment was defined as the relief of clinical symptoms or a reduction in lesions, as indicated by imaging studies. Poor treatment efficacy was defined as the absence of improvement or deterioration of clinical symptoms and imaging findings following treatment.

### 2.5. Ethical Considerations

This study was performed in accordance with the principles of the Declaration of Helsinki and approved by the Ethics Committee of West China Hospital, Sichuan University (approval number: 2020-48). The requirement for informed consent was waived due to the noninterventional nature of the study.

### 2.6. Statistical Analysis

Statistical analyses were performed using IBM SPSS Statistics version 26.0 (IBM Corp., Armonk, NY, USA). Categorical variables were compared using the χ^2^ test or Fisher’s exact test. Continuous variables following a normal distribution were reported as mean ± standard deviation and compared using Student’s *t*-test. Skewed continuous variables were presented as median and interquartile range (IQR), with group comparisons conducted using the Mann–Whitney U test. Logistic regression modeling was applied to identify risk factors associated with PR-CRE infections. Survival analysis was performed using the Kaplan–Meier (KM) method and Cox regression analysis. *p*-values < 0.05 were considered statistically significant.

## 3. Results

### 3.1. Patient Characteristics

The PR-CRE group included 127 patients, comprising 89 males and 38 females. Among them, 122 patients were infected with *Klebsiella pneumoniae*, three patients were diagnosed with *Escherichia coli* infections, and two patients were diagnosed with *Enterobacter cloacae* infections. Pulmonary infections were the most prevalent (53.54%), with a CD4+ T cell count of 200.0 (124.0, 363.0) and a 28-day all-cause mortality rate of 33.02%. Following propensity score-matching rules, the PS-CRE group comprised 127 patients, which served as the control group (Table 1).

### 3.2. Antimicrobial Susceptibility Testing

Both ceftazidime–avibactam and tigecycline exerted effective antibacterial effects against PR-CRE and PS-CRE. Nevertheless, tigecycline demonstrated higher sensitivity (≥94.4%) compared to ceftazidime–avibactam (≥81.5%). A significant difference was observed in the distribution of the tetracycline susceptibility test results between the PR-CRE and PS-CRE groups (*p* = 0.029). Notably, the sensitivity rates of PR-CRE strains to amikacin and cotrimoxazole were 29.1% and 24.4%, respectively (Table 2).

### 3.3. Risk Factor Analysis for PR-CRE Infections

Factors such as pre-sampling hospital stay duration, chronic diseases at sampling sites, history of invasive procedures, immune status, and antimicrobial exposure were incorporated in the risk factor analysis. Univariate analysis revealed that prolonged hospitalization; chronic organic diseases; immunosuppression; organ transplantation; and ≥3 days of use of antimicrobials, including aminoglycosides, cotrimoxazole, carbapenems, tigecycline, ceftazidime–avibactam, and polymyxin, were significant risk factors for PR-CRE infection (*p* < 0.05). Meanwhile, multivariate analysis identified the presence of chronic organic diseases (OR, 2.747; 95% CI, 1.303–5.789; *p* = 0.008) and the use of polymyxin ≥ 3 d (OR, 19.203; 95% CI, 7.126–51.752; *p* < 0.001) as independent risk factors for PR-CRE infection (Table 3).

### 3.4. Treatment Efficacy in PR-CRE Patients

**Figure 1:**
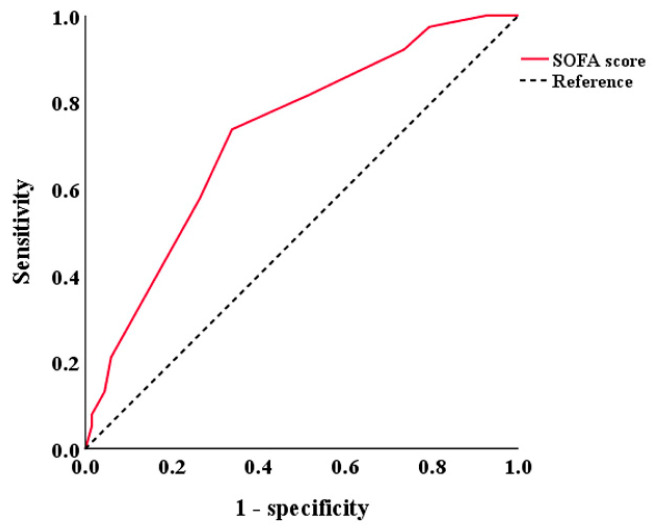
Receiver operating characteristic curve of SOFA score for predicting treatment outcomes in patients with polymyxin- and carbapenem-resistant Enterobacteriaceae infections.

Among the 127 patients with PR-CRE infection, 106 patients received the specified treatment regimen and were included in the analysis, whereas the remaining 21 cases were patients who did not receive treatment; had a treatment duration of fewer than 3 days; or were administered other treatment regimens, such as the combination of carbapenem and aminoglycosides. Considering that the SOFA score is widely used to assess infection severity, and since univariate analysis indicated a significant association between SOFA scores and clinical outcomes (Z = −3.839, *p* < 0.001), patients were stratified based on the optimal SOFA-score cut-off value (SOFA = 5.5; 95% CI, 0.724 (0.625–0.823); *p* < 0.001; Figure 1).

The treatment efficacy rates for the tigecycline group, the ceftazidime–avibactam group, and the combination treatment group were 58.46%, 71.43%, and 76.92%, respectively. In the subgroup analysis, the ceftazidime–avibactam group consistently demonstrated higher efficacy compared to the tigecycline group (90.91% vs. 76.32%; 58.52% vs. 33.33%), although the difference was not statistically significant (*p* > 0.05). In the SOFA score ≥ 5.5 subgroup, the treatment efficacy of combination therapy was comparable to ceftazidime–avibactam (57.14% vs. 58.82%, *p* > 0.05; Table 4).

Additionally, 11 isolates exhibited resistance to ceftazidime–avibactam. Of these, three patients were treated with ceftazidime–avibactam and aztreonam, achieving favorable responses in two cases. The remaining eight patients were treated with tigecycline-based regimens, with three patients displaying favorable responses.

### 3.5. Twenty-Eight-Day In-Hospital Mortality Analysis for Patients with PR-CRE Infection

The 28-day all-cause mortality rate among PR-CRE-infected patients was 33.02% (35/106). Univariate analysis indicated that older age, bloodstream infection, aCCI ≥ 2.5, and SOFA score ≥ 5.5 were associated with 28-day all-cause mortality in patients with PR-CRE infection (*p* < 0.05). Multivariate analysis identified a SOFA score ≥ 5.5 (HR, 6.718; 95% CI, 2.526–17.866; *p* < 0.001) as an independent risk factor for 28-day all-cause mortality in patients with PR-CRE infection (Table 5).

**Figure 2:**
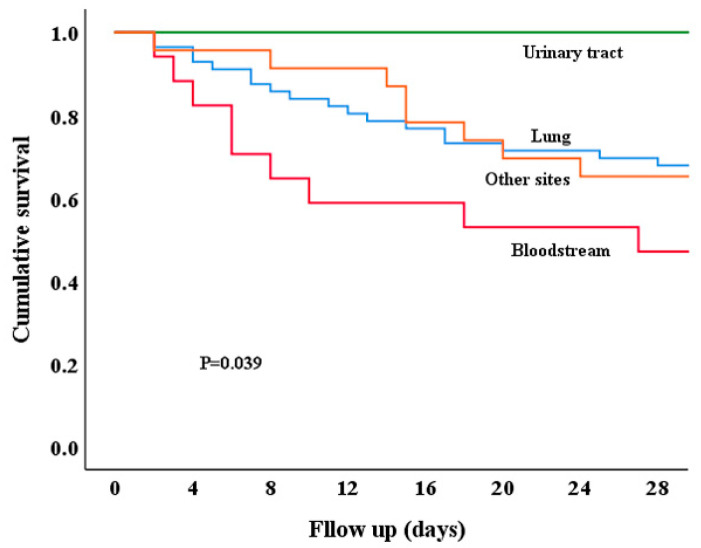
Analysis of 28-day survival in patients with polymyxin- and carbapenem-resistant Enterobacteriaceae infection at different sites. Kaplan–Meier survival analysis demonstrated that the survival rates of patients with different infection sites varied significantly (*p* = 0.039). Upon pairwise comparisons, it was found that patients with urinary tract infections had a higher survival rate compared to those with bloodstream (*p* = 0.007) or lung infections (*p* = 0.008), and there was no statistical difference among other groups (*p* > 0.05).

The KM survival analysis illustrated that patients with urinary tract infections had the highest survival rates, whereas those with bloodstream infections had the lowest survival rates (*p* = 0.039; Figure 2). Notably, although the 28-day survival rate for patients with urinary tract infections was the highest, three patients died after the 28 days.

**Figure 3:**
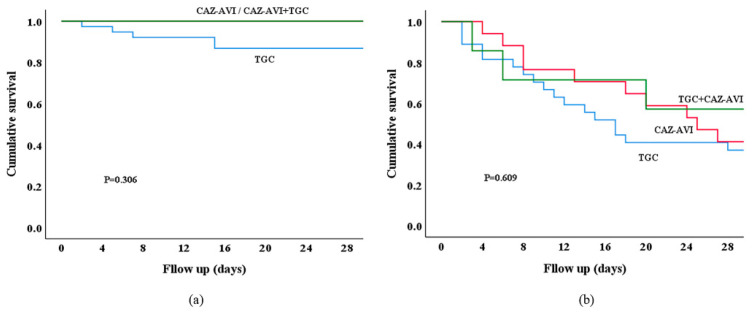
Analysis of 28-day survival in patients with polymyxin- and carbapenem-resistant Enterobacteriaceae (PR-CRE) infection treated with different regimens. (**a**) Patients with SOFA score < 5.5 were divided into three groups according to different antimicrobial regimens: ceftazidime–avibactam (CAZ-AVI)-based regimen (*n* = 11), tigecycline (TGC)-based regimen (*n* = 38), and CAZ-AVI combined with TGC group (*n* = 6). Survival analysis showed no statistically significant difference in the 28-day survival rate among PR-CRE-infected patients (*p* = 0.306). (**b**) Patients with SOFA score ≥ 5.5 were divided into three groups according to different antimicrobial regimens: ceftazidime–avibactam (CAZ-AVI)-based regimen (*n* = 17), tigecycline (TGC)-based regimen (*n* = 27), and CAZ-AVI combined with TGC group (*n* = 7). Survival analysis showed no statistically significant difference in the 28-day survival rate among PR-CRE-infected patients (*p* = 0.609).

The 28-day survival rates among patients treated with ceftazidime–avibactam, tigecycline, and combination therapy were comparable, regardless of SOFA score < 5.5 (*p* = 0.306) (Figure 3a) or SOFA score ≥ 5.5 (*p* = 0.609) (Figure 3b).

## 4. Discussion

Enterobacteriaceae are common pathogens for hospital-acquired infections, with CRE posing a severe threat to human health. Despite the use of novel antimicrobials such as ceftazidime–avibactam, meropenem–vaborbactam, and cefiderocol for clinical use, they remain inaccessible in several countries and regions, with polymyxins still being used as the last line against carbapenem-resistant Gram-negative bacterial infections. However, the growing issue of polymyxin resistance among Gram-negative bacteria is concerning.

Herein, the case group primarily consisted of *Klebsiella pneumoniae*, with a small proportion of other Enterobacteriaceae species. Previous studies have reported similar patterns of microbial distribution [15,16,17]. This distribution was likely influenced by *Escherichia coli* and *Klebsiella pneumoniae* being the top two species isolated from clinical samples, with the latter exhibiting a significantly higher polymyxin resistance rate compared to *Escherichia coli* (11.8% vs. 1.5%) [18]. In this study, both PR-CRE and PS-CRE were highly sensitive to tigecycline and ceftazidime–avibactam. Despite sensitivity to amikacin being relatively low, it remained higher than that of other β-lactam and quinolone antibiotics, positioning it as a viable option for combination therapy. A previous study documented that aminoglycosides protected against mortality from polymyxin-resistant strains [15]. Additionally, guidelines recommend combination therapies that include amikacin or other aminoglycosides for the treatment of CRE infections [19].

Multivariate analysis revealed that the presence of chronic organic disease and the use of polymyxin were independent risk factors for the occurrence of PR-CRE infection in hospitalized patients. Chronic organic diseases, especially chronic lung diseases, generally involve local mucosal barrier damage or dysfunction, which facilitates pathogen colonization and subsequent infection [20,21,22]. Colonization increases the risk of infection, thereby limiting clearance from the corresponding sites [23]. It is worthwhile emphasizing that earlier studies have also identified chronic obstructive pulmonary disease (COPD) as an independent risk factor for polymyxin-resistant Acinetobacter baumannii bloodstream infections in patients without a history of polymyxin exposure [24]. As common pathogens responsible for hospital-acquired infections, the members of the Enterobacteriaceae family are more likely to colonize or infect these sites. The polymyxin exposure and improper use exert selective pressure on bacteria, thereby increasing the likelihood of inducing resistance mutations and leading to polymyxin resistance. A retrospective study enrolling 166 PSKP (polymyxin-sensitive *Klebsiella pneumoniae*)-infected patients treated with polymyxin concluded that low-dose, prolonged polymyxin treatment may induce resistance mutations in *Klebsiella pneumoniae* [25]. Likewise, another study signaled that polymyxin exposure-induced resistance mutations (*mgrB*, *phoQ*, *crrB*, *pmrB*, *pmrA*, and *phoP*) were the chief reason underlying polymyxin resistance in most patients and that polymyxin MIC was correlated with the number of affected resistance genes [26]. The poor distribution of polymyxins in the lungs following intravenous administration may promote resistance to polymyxins [27]. Therefore, it is crucial to highlight the need for optimizing polymyxin utilization as a strategy to prevent resistance. In addition, plasmids carrying the *mcr* gene significantly promoted bacterial polymyxin resistance, which was also observed in clinical Enterobacteriaceae isolates [28,29,30,31]. The potential for horizontal gene transfer has raised significant concerns about the dissemination of resistance genes. Farm animals and aquaculture serve as reservoirs for the *mcr* gene, likely due to the widespread use of polymyxins in animal husbandry for growth promotion, infection prevention, and treatment [3,32,33]. With the reintroduction of polymyxins in clinical practice, this issue has become increasingly concerning [34]. Fortunately, many countries have approved the withdrawal of polymyxins as a feed additive for animals [35,36,37]. However, continuous monitoring remains essential.

Although the efficacy analysis demonstrated no statistically significant difference between ceftazidime–avibactam-based regimens and tigecycline-based regimens, this may be attributed to the limited sample size in the ceftazidime–avibactam group, a common limitation in real-world studies. Nevertheless, we observed a consistent trend toward higher efficacy with ceftazidime–avibactam, and this trend aligns with previous studies and clinical guidelines recommending ceftazidime–avibactam as a preferred therapeutic option for CRE infections, including polymyxin-resistant strains [19,38,39]. Low plasma levels of tigecycline may account for its suboptimal efficacy. Importantly, the FDA has issued safety warnings indicating that the mortality rate among patients receiving tigecycline for the treatment of hospital-acquired pneumonia (HAP) is on the rise, prompting the addition of a black box warning to the tigecycline label [40]. Additionally, tigecycline is generally recommended for use in combination with polymyxins, carbapenems, or aminoglycosides, and inappropriate combination regimens may affect the efficacy of tigecycline. Furthermore, our results uncovered that the combination of ceftazidime–avibactam with tigecycline did not significantly improve efficacy in patients with PR-CRE infections compared to the ceftazidime–avibactam regimen alone, suggesting that combination therapy may be unnecessary. Klebsiella pneumoniae carbapenemase (KPC) is the most prevalent carbapenemase among Enterobacteriaceae, particularly among *Klebsiella pneumoniae* isolates worldwide. In China, the proportion of CRKP producing KPC can reach up to 90.9%, with KPC-2 being the predominant variant [41]. In this study, the CAZ-AVI regimen showed favorable clinical efficacy in both PR-CRE and PS-CRE groups, which was likely due to the high proportion of CRKP isolates and the prevalence of KPC-producing strains in China. Notably, CAZ-AVI demonstrates no in vitro activity against metallo-β-lactamase (MBL)-producing CRE. These resistant strains are highly prevalent in multiple countries, including Egypt, India, Korea, Mexico, Russia, and Thailand, with New Delhi metallo-β-lactamase (NDM) representing the predominant genotype [42]. Current guidelines recommend the combination of CAZ-AVI with aztreonam as the preferred therapeutic option for MBL-producing CRE infections [19]. Phenotypic or genotypic characterization of carbapenemase production through laboratory testing represents a reliable approach to guide optimal antimicrobial therapy.

Previous studies have reported 28-day all-cause mortality rates of approximately 50% in patients with PR-CRE infection and up to 66% in patients with bloodstream infections, which is higher than the results of the present study [11,13]. This discrepancy may be attributed to the significant impact of comorbidities on all-cause mortality and the limited sample sizes in prior studies, many of which primarily enrolled hospitalized patients from before 2018. In recent years, advancements in diagnostic techniques have enabled the prompt identification of pathogens in clinical settings. Furthermore, the development of antimicrobial agents has significantly contributed to the reduction of 28-day mortality rates among these patients. Cox survival analysis identified a SOFA score ≥ 5.5 as an independent risk factor for 28-day mortality in patients infected with PR-CRE. Similarly, a retrospective cohort study involving 184,875 patients highlighted the strong association between SOFA scores and in-hospital mortality in critically ill patients with infections [43]. Timely and regular assessment of SOFA scores may potentially enable doctors to promptly tailor treatment strategies, potentially enhancing patient outcomes. This underscores the importance of integrating SOFA score monitoring into clinical practice for the management of PR-CRE-infected patients, aiding in timely adjusting therapeutic approaches based on real-time clinical data.

Survival analysis indicated that patients with urinary tract infections had the highest 28-day survival rate, while those with bloodstream infections had the lowest survival rate. While research on the prognosis of patients with PR-CRE infections at different sites is limited, similar findings have been reported in CRE infections [44,45]. This discrepancy may be related to the severity and rapid progression of bloodstream infections, which are more prone to result in multiorgan dysfunction, making timely intervention and treatment more challenging. In addition, numerous studies have established that inappropriate empiric therapy is associated with higher mortality rates in patients with bloodstream infections, highlighting the importance of rapid and accurate pathogen identification [46,47,48].

Nevertheless, some limitations of this study cannot be overlooked. To begin with, this was a single-center retrospective study. The sample sizes for certain subgroups, such as those with different treatment regimens or infection sites, were relatively small, which may have compromised the generalizability of our findings. Furthermore, our study lacked molecular characterization of the resistant isolates. Without whole-genome sequencing (WGS) or PCR-based resistance-gene profiling, we could not determine the underlying mechanisms of resistance (e.g., *blaKPC*, *blaNDM*, *mcr*, or porin mutations). Additionally, sample heterogeneity and incomplete documentation of treatment regimen details are also unavoidable limitations of this study.

## 5. Conclusions

The presence of chronic organic disease and the polymyxin use for ≥3 days were identified as independent risk factors for PR-CRE infection among patients. Optimizing the administration strategy of polymyxin may be a key measure to mitigate the risk of polymyxin resistance in CRE. Ceftazidime–avibactam-based regimens may be an effective option for the treatment of PR-CRE infection. Finally, a SOFA score ≥ 5.5 was associated with a poor prognosis in patients with PR-CRE infection.

## Figures and Tables

**Table 1:** Characteristics of patients with polymyxin- and carbapenem-resistant Enterobacteriaceae infections.

Variables	PR-CRE Group (*n* = 127)	PS-CRE Group (*n* = 127)	Statistical Values	*p*-Value
Demographic variables				
Age	(55.70 ± 16.80)	(57.62 ± 16.10)	-	-
Male	70.08% (89/127)	69.29% (88/127)	-	-
Bacterial species				
*Klebsiella pneumoniae*	96.06% (122/127)	96.06% (122/127)	-	-
*Escherichia coli*	2.36% (3/127)	2.36% (3/127)	-	-
*Enterobacter cloacae*	1.57% (2/127)	1.57% (2/127)	-	-
Laboratory test results				
White blood cell (WBC) (10^9^/L)	10.25 (6.64, 16.55)	9.28 (6.74, 13.57)	Z = −0.635	0.525
Neutrophil count (10^9^/L)	8.09 (5.16, 14.08)	7.92 (5.23, 11.54)	Z = 0.062	0.950
Procalcitonin (PCT) (ng/mL)	1.11 (0.34, 3.45)	0.79 (0.19, 4.11)	Z = −1.199	0.230
Interleukin-6 (IL-6) (pg/mL)	80.24 (29.15, 288.60)	76.10 (21.90, 301.90)	Z = −0.053	0.958
C-reactive protein (CRP) (mg/L)	75.20 (25.70, 152.00)	77.40 (33.80, 118.00)	Z = −0.516	0.606
CD3+ T lymphocyte (cell/µL) **^a^**	378.5 (224.0, 599.0)	414.5 (233.0, 633.0)	Z = 0.191	0.848
CD4+ T lymphocyte (cell/µL) **^a^**	200.0 (124.0, 363.0)	241.5 (116.5, 375.0)	Z = 0.702	0.483
CD8+ T lymphocyte (cell/µL) **^a^**	137.0 (61.0, 276.0)	151.0 (63.5, 261.5)	Z = −0.293	0.769
Lymphocyte count (cell/µL) **^b^**	0.74 (0.48, 1.095)	0.8 (0.47, 1.08)	Z = 0.272	0.786
Infection sites				
Lung	53.54% (68/127)	59.06% (75/127)	χ^2^ = 0.784	0.376
Bloodstream	18.9% (24/127)	14.96% (19/127)	χ^2^ = 0.700	0.403
Urinary tract	7.87% (10/127)	8.66% (11/127)	χ^2^ = 0.052	0.820
Other sites **^c^**	19.69% (25/127)	17.32% (22/127)	χ^2^ = 0.235	0.628
28-day all-cause mortality ^**d**^	33.02% (35/106)	35.78% (42/109)	χ^2^ = 0.711	0.399

**^a^** PR-CRE group (*n* = 74); PS-CRE group (*n* = 48). **^b^** PR-CRE group (*n* = 120); PS-CRE group (*n* = 109). **^c^** Including intracranial, incision, thoracic cavity, and abdominal cavity. **^d^** Twenty-eight-day all-cause mortality in patients treated with “sensitive” antibiotics according to susceptibility results post-infection (treatment duration ≥ 3 days). PR-CRE: polymyxin-resistant and carbapenem-resistant Enterobacteriaceae; PS-CRE: polymyxin sensitive and carbapenem-resistant Enterobacteriaceae

**Table 2:** Antimicrobial susceptibility testing results of carbapenem-resistant Enterobacteriaceae isolates.

Antimicrobial Agents	PR-CRE Group (*n* = 127)			PS-CRE Group (*n* = 127)			*p*-Value
	S	I	R	S	I	R	
	% (No. of Susceptible Isolates/no. of Isolates Tested)						
Amikacin	29.1 (37/127)	1.6 (2/127)	69.3 (88/127)	23.6 (30/127)	1.6 (2/127)	74.8 (95/127)	0.605
Ceftazidime	0	0	100 (127/127)	0	0	100 (127/127)	-
Ceftazidime–avibactam	88.9 (104/117)	0	11.1 (13/117)	81.5 (97/119)	0	18.5 (22/119)	0.111
Levofloxacin	0	1.6 (2/127)	98.4 (125/127)	0.8 (1/127)	0	99.2 (126/127)	0.498
Meropenem	1.6 (2/127)	0	98.4 (125/127)	0.8 (1/127)	0.8 (1/127)	98.4 (125/127)	1.00
Minocycline	9.5 (10/105)	19 (20/105)	71.5 (75/105)	16.5 (17 /103)	16.5 (17/103)	67 (69/103)	0.324
Co-trimoxazole	24.4 (31/127)	1.6 (2/127)	74 (94/127)	16.5 (21/127)	3.1 (4/127)	80.4 (102/127)	0.23
Tetracycline	7.9 (10/126)	4 (5/126)	88.1 (111/126)	13.4 (17/127)	0	86.6 (110/127)	0.029
Tigecycline	94.4 (117/124)	4.8 (6/124)	0.8 (1/124)	94.5 (120/127)	64.7 (6/127)	0.8 (1/127)	1.00
Piperacillin–tazobactam	0	0.8 (1/127)	99.2 (126/127)	0	0	100 (127/127)	1.0

PR-CRE, polymyxin-resistant and carbapenem-resistant Enterobacteriaceae; PS-CRE, polymyxin sensitive and carbapenem-resistant Enterobacteriaceae; S, susceptible; I, intermediate; R, resistance.

**Table 3:** Risk factor analysis for polymyxin- and carbapenem-resistant Enterobacteriaceae infections.

Variables	Univariate Analysis			Multivariate Analysis	
	PR-CRE Group (*n* = 127)	PS-CRE Group (*n* = 127)	*p*-Value	OR (95% CI)	*p*-Value
Vulnerability factors					
Length of hospital stay	34.0 (20.5, 53.0)	23.0 (12.0, 31.0)	<0.001	0.99 (0.972, 1.008)	0.268
Consciousness disorders	82	86	0.596		
Invasive procedure of sampling sites **^a^**	116	106	0.059		
Body Mass Index (kg/m^2^)	22.86 (20.2, 25.95)	23.48 (21.01, 25.64)	0.373		
Uncontrolled diabetes mellitus	37	28	0.196		
Chronic organic diseases at sampling sites **^b^**	43	18	<0.001	2.747 (1.303, 5.789)	0.008
Immunosuppression	36	15	0.001	2.298 (0.864, 6.112)	0.096
Organ transplantation	19	8	0.025	1.237 (0.358, 4.268)	0.737
Intensive care unit	102	94	0.232		
Operation	65	75	0.207		
Antimicrobial exposure					
Beta-lactamase inhibitors	105	114	0.101		
Cephalosporins	38	41	0.547		
Quinolones	24	25	0.874		
Aminoglycosides	17	7	0.032	0.971 (0.280, 3.368)	0.963
Co-trimoxazole	12	4	0.039	0.673 (0.129, 3.514)	0.639
Carbapenems	99	80	0.009	0.981 (0.482, 1.997)	0.958
Tigecycline	72	36	<0.001	1.114 (0.536, 2.314)	0.773
Ceftazidime–avibactam	10	1	0.006	1.995 (0.165, 24.159)	0.587
Polymyxin	67	6	<0.001	19.203 (7.126, 51.752)	<0.001

PR-CRE, polymyxin-resistant and carbapenem-resistant Enterobacteriaceae; PS-CRE, polymyxin sensitive and carbapenem-resistant Enterobacteriaceae; OR, odds ratio; 95% CI, 95% confidence interval. **^a^** Including local surgery, endoscopy, tracheal intubation, and central venous catheterization. **^b^** Including chronic obstructive pulmonary disease, bronchiectasis, biliary calculi, urinary tract stones, and chronic pyelonephritis.

**Table 4:** Efficacy analysis of different treatment regimens in patients with polymyxin- and carbapenem-resistant Enterobacteriaceae infections by severity level.

	Tigecycline Treatment Group ^b^ (*n* = 65)	Ceftazidime–Avibactam Treatment Group ^c^ (*n* = 28)	Combination Treatment Group ^d^ (*n* = 13)	*p*-Value
Treatment efficacy rate (*n* = 106) **^a^**	58.46% (38/65)	71.43% (20/28)	76.92% (10/13)	0.289
SOFA score < 5.5 (*n* = 55)	76.32% (29/38)	90.91% (10/11)	100.00% (6/6)	0.342
SOFA score ≥ 5.5 (*n* = 51)	33.33% (9/27)	58.82% (10/17)	57.14% (4/7)	0.192

SOFA, Sequential Organ Failure Assessment. **^a^** Including 104 patients with *Klebsiella pneumoniae* infection and 2 patients with *Escherichia coli* infection; **^b^** tigecycline monotherapy and tigecycline-based regimens; **^c^** ceftazidime–avibactam monotherapy and ceftazidime–avibactam-based regimens; **^d^** tigecycline combined with ceftazidime–avibactam.

**Table 5:** Cox regression analysis of 28-day in-hospital mortality for patients with polymyxin- and carbapenem-resistant Enterobacteriaceae infection.

Variables	Univariate Analysis		Multivariate Analysis	
	HR (95% CI)	*p*-Value	HR (95% CI)	*p*-Value
Demographic variables				
Male	1.099 (0.538, 2.244)	0.796		
Age	1.024 (1.003, 1.045)	0.025	1.012 (0.988, 1.036)	0.343
Infection sites				
Lung	0.941 (0.485, 1.825)	0.856		
Bloodstream	2.298 (1.076, 4.911)	0.032	1.842 (0.816, 4.158)	0.141
Urinary tract	0.041 (0, 4.038)	0.173		
Other sites	1.014 (0.461, 2.233)	0.972		
Vulnerability factors				
SOFA scores ≥ 5.5	8.756 (3.388, 22.632)	<0.001	6.718 (2.526, 17.866)	<0.001
aCCI ≥ 2.5	3.389 (1.195, 9.606)	0.022	1.799 (0.576, 5.619)	0.312
Uncontrolled diabetes mellitus	1.324 (0.658, 2.663)	0.431		
Chronic kidney disease	0.675 (0.295, 1.547)	0.353		
Chronic organic diseases of infection sites	0.697 (0.341, 1.422)	0.321		
Immunosuppression	0.949 (0.445, 2.026)	0.893		
Organ transplantation	0.801 (0.311, 2.065)	0.646		
History of surgical procedures before infection	0.823 (0.374, 1.813)	0.629		
Treatment				
Tigecycline	1.139 (0.574, 2.261)	0.710		
Ceftazidime–avibactam	1.055 (0.507, 2.198)	0.885		
Tigecycline and ceftazidime–avibactam	0.642 (0.197, 2.097)	0.463		

PR-CRE, polymyxin- and carbapenem-resistant Enterobacteriaceae; HR, hazard ratio; SOFA, Sequential Organ Failure Assessment; aCCI: age-adjusted Charlson Comorbidity Index.

## Data Availability

The original contributions presented in this study are included in the article. Further inquiries can be directed to the corresponding author.

## Supplementary Materials

The following supporting information can be downloaded at https://www.mdpi.com/article/10.3390/microorganisms13061256/s1, Table S1: MIC Breakpoints for Enterobacterales.
