# Supplementary material for: Risk Factors and Prognosis of Polymyxin- and Carbapenem-Resistant Enterobacteriaceae Infections: A Propensity-Matched Real-World Study
Source: Microorganisms. 2025 May 29;13(6):1256. doi: 10.3390/microorganisms13061256 (PMC12194920; doi:10.3390/microorganisms13061256)
Supplement: Supplementary file 1 [file microorganisms-13-01256-s001.zip › microorganisms-3624854-supplementary.pdf]

Table S1. MIC Breakpoints for Enterobacterales

**Antimicrobial agent**	Interpretive Categories and MIC Breakpoints（μg/mL）		
	S	I	R
Amikacin	⩽ 16	32^	⩾ 64
Ceftazidime	⩽ 4	8^	⩾ 16
Levofloxacin	⩽ 0.5	1^	⩾ 2
Meropenem	⩽ 1	2	⩾ 4
Minocycline	⩽ 4	8	⩾ 16
Co-trimoxazole	⩽ 2/38	-	⩾ 4/76
Tetracycline	⩽ 4	8	⩾ 16
Tigecycline	⩽ 2	4	⩾ 8
Piperacillin-tazobactam	⩽ 16/4	32/4–64/4^	⩾ 128/4

Intermediate ranges denoted with a “^” for the applicable antimicrobial agents.
